# What outcomes matter in recovery from depression? A pilot study of young adults

**DOI:** 10.20935/MHealthWellB8342

**Published:** 2026-06-19

**Authors:** Jeffrey A. Lam, Norah Mulvaney-Day, Alexandra R. Dobbins, Rajendra Aldis, Ishan Pasricha, Hermioni L. Amonoo, Ana M. Progovac

**Affiliations:** 1 Department of Psychiatry, Cambridge Health Alliance, Cambridge, MA, USA.; 2 Department of Psychiatry, Harvard Medical School, Boston, MA, USA.; 3 Department of Supportive Oncology, Dana-Farber Cancer Institute, Boston, MA, USA.; 4 Department of Psychiatry, Brigham and Women’s Hospital, Boston, MA, USA.; 5 Institute for Excellence in Health Equity, New York University Grossman School of Medicine, New York, NY, USA.; 6 Department of Population Health, New York University Grossman School of Medicine, New York, NY, USA.

**Keywords:** major depressive disorder, personal recovery, flourishing, psychological well-being, patient-centered outcomes

## Abstract

**Introduction:**

“Positive” outcomes such as flourishing and personal recovery are important predictors of physical health, mental illness, quality of life, and all-cause mortality. However, behavioral health outcome measures often emphasize symptom reduction, while overlooking “positive” outcomes. The objective of this study is to describe (1) the outcomes most important to individuals with major depressive disorder (MDD) and (2) “positive” outcomes and psychiatric symptoms in a real-world sample of young adults with MDD.

**Materials and methods:**

Forty-three participants aged 18–35 with a diagnosis of MDD receiving outpatient behavioral health treatment in an urban safety-net psychiatry department completed online surveys identifying factors most important to recovery and standardized measures of flourishing, personal recovery, functioning, and symptoms of depression and anxiety.

**Results:**

Most participants (61.9%) had high symptoms of depression (Patient Health Questionnaire-8 (PHQ-8) ≥ 10), 16.7% were flourishing (Flourishing Scale ≥ 48), and 41.9% had high personal recovery (Brief INSPIRE-O ≥ 50). Factors ranked as “most important” for personal recovery included coping well with stressful events (39.5%), functioning well (34.9%), and having hopes and dreams for the future (30.2%). Notably, among individuals with low depressive symptoms, 60.0% reported low flourishing and 31.3% reported low personal recovery.

**Conclusions:**

Individuals with MDD value a diverse range of outcomes, and exhibit a large variation in levels of depressive symptoms and characteristics of “positive” mental health. Aligning outcome tracking with patient-defined goals may provide a more comprehensive understanding of overall mental health.

## 1. Introduction

Behavioral healthcare often prioritizes symptomatic remission when measuring treatment outcomes, sometimes overlooking other important outcomes. Within treatment of major depressive disorder (MDD), researchers commonly define “response” as a ≥ 50% reduction in depression symptoms and “remission” as scores below a certain threshold [1]. This symptom-focused approach reflects the medical model of illness and prioritizes return to previous levels of functioning and symptoms [2]. As behavioral health systems continue to adopt measurement-based care, defined as the routine use of validated patient-reported outcomes to inform diagnosis, monitor progress, and guide treatment decisions [3], there remains a critical need to better understand which outcomes are most important to measure [4].

The emerging literature shows that patients prioritize outcomes beyond symptomatic reduction [5, 6], and there is a growing movement to measure more “positive” mental health outcome measures [7]. While there is yet to be a consensus on “positive” mental health assessment instruments or even well-established definitions [8], the constructs of flourishing [9-11] and personal recovery [12] are two emerging constructs that operationalize and capture broader dimensions of well-being that extend beyond symptoms. “Flourishing” is a multidimensional outcome measure that is broadly understood as an evolving state of optimal well-being and functioning across multiple domains of an individual’s life, including emotional, psychological, and social functioning [9-11]. Personal recovery emerged from perspectives of individuals with lived experience of mental illness [2] and can be defined by the idiosyncratic process of changing one’s attitude, value, feelings, and goals, and developing meaning in life beyond illness. Personal recovery is characterized by connection, hope, identity, meaning, and empowerment [12].

Assessing “positive” outcomes, such as flourishing and personal recovery, may have benefits for patient and population health. First, defining “positive” goals may mitigate stigma and improve help-seeking behavior [13]. Secondly, service users and clinicians tend to focus on different treatment outcomes [5]. Understanding the outcomes that service users care most about may improve the therapeutic relationship between clinicians and service users [14]. Lastly, from a population health perspective, assessing flourishing and recovery outcomes may offer a more comprehensive way to characterize overall health [15, 16]. A budding body of research suggests that overall mental health should be characterized by two dimensions: “positive” mental health and symptoms of mental illness. Instead of a single continuum from mental illness to mental well-being, in a “dual-continua” model of mental health, individuals can be classified into four states: (A) high “positive” mental health without mental illness, (B) high “positive” mental health with mental illness, (C) low “positive” mental health without mental illness, and (D) low “positive” mental health with mental illness [15, 17]. Despite these potential benefits, few mental health research studies and few mental health clinicians assess “positive” outcomes such as flourishing and personal recovery [7, 18].

Few studies have described the mental health outcomes most important for individuals with MDD in young adult samples [5, 19, 20]. Young adults are a particularly important population to examine given early adulthood is the peak period for both onset and prevalence of MDD and a critical period for identity formation and goal-setting [21, 22]. Moreover, despite considerable conceptual overlap between “positive” mental health assessment instruments, little is known about how these outcome measures relate to each other in real-world clinical samples. In this study, conducted in a diverse, safety-net outpatient community-based practice, we aimed to describe (1) the recovery outcomes most important to service users and (2) measures of personal recovery, measures of flourishing, and psychiatric symptoms in a real-world sample of young adults with MDD.

## 2. Materials and methods

### 2.1. Eligibility criteria

Data were collected from a sample of young adults recruited from general outpatient psychiatric clinics at an academic public safety-net system that serves a diverse population of approximately 140,000 service users annually [23]. We restricted our sample to participants aged 18–35 to focus on young adults. Eligible participants had a documented diagnosis of MDD and were in active outpatient treatment, defined as having a psychiatry or therapy appointment with the outpatient psychiatry department between January and July, 2024. Additional eligibility requirements included: English fluency determined by phone screening by the research coordinator, access to the internet, and having a device capable of completing the REDCap survey (e.g., computer, smartphone, or tablet). Individuals with an electronic health record diagnosis of schizophrenia spectrum disorder or bipolar disorder were excluded from participation in order to focus on the unique treatment goals of individuals with MDD.

### 2.2. Recruitment

An Institutional Review Board approved the research protocol (CHA-IRB-23-24-308). A list of 505 potential participants was pulled from electronic health records, using the inclusion and exclusion criteria defined above. A research coordinator attempted to call and/or email 359 potentially eligible participants from October 2024 to February 2025. We were unable to reach 229 participants and 62 declined to participate. For the remaining 68 individuals, we verbally confirmed eligibility by asking about age, current behavioral health treatment, and race/ethnicity prior to invitation to participate in the study. After confirming eligibility, participants reviewed an electronic informed consent form with a research assistant present to confirm understanding. All participants provided informed consent by verbal agreement and an electronic signature via the REDCap survey platform prior to beginning the study questionnaire. Survey data collection took place during the same time period from October 2024 to February 2025. All participants received a $35 gift card on an online platform for completing the survey. See Figure S1 for an overview of the recruitment process.

### 2.3. Data collection

A total of 55 individuals were emailed the survey after a verbal screening process, out of whom 45 individuals completed the survey. Two individuals were excluded during the post-survey data quality check (one for not meeting eligibility criteria and another for inattentive survey response patterns). A total of 43 surveys were included for analysis.

We used a cross-sectional survey design, whereby participants were administered a survey in English at a single time point. The survey contained questions about positive psychological well-being (Flourishing Scale, Secure Flourishing Measure), recovery (Brief INSPIRE-O and Questionnaire about the Process of Recovery (QPR)), functioning (Work and Social Adjustment Scale (WSAS)), mental illness symptoms (Patient Health Questionnaire-8, Generalized Anxiety Disorder-7), sociodemographic information (age, sex, gender identity, relationship status, race/ethnicity, history of depression and recovery), and outcomes most important in recovery. We used two recovery measures and two flourishing measures because we could not identify prior studies that examined how these measures relate to one another in individuals with MDD. Measures were selected based on validity, reliability, ease of administration, and accessibility. The measures used are described in detail in Table 1.

### 2.4. Statistical analysis

We used Stata version 18.5 (StataCorp, College Station, TX, USA) for all analyses. We first summarized participants’ demographics, symptoms of mental illness, flourishing, and personal recovery with descriptive statistics: mean and standard deviation for continuous variables and proportions for categorical variables. We then described these same participant characteristics by low and high depressive symptoms (Patient Health Questionnaire-8 (PHQ-8) ≥ 10). A PHQ-8 score of ≥ 10 has an 88% sensitivity and 88% specificity for major depression, and, regardless of diagnostic status, typically represents clinically significant depression [33, 34].

Next, we summarized the factors participants rated as most and least important for recovery using means and standard deviations of Likert scale items and the proportion selecting each factor as “Most Important.” We then described these same factors by low and high depressive symptoms (PHQ-8 ≥ 10), and used t-tests to evaluate for any differences between high and low depressive symptom groups. We then summarized the Spearman correlation coefficients between all continuous measures (e.g., flourishing, recovery, mental illness measures, functioning) to evaluate the strength and directionality of the relationships between these constructs.

Lastly, we created scatterplots to visually explore the relationship between (1) flourishing and depressive symptoms and (2) recovery and depressive symptoms, consistent with the dual-continua model of mental health. We used the Flourishing Scale for flourishing and Brief INSPIRE-O for personal recovery for this analysis because these two measures have validated cutoff points in the literature, allowing us to divide the scatterplot into quadrants consistent with the dual-continua model [35]. We used complete case analysis for all analyses. One participant had missing PHQ-8 data and another participant had missing Flourishing Scale data, resulting in slight variation in sample sizes across analyses.

## 3. Results

Table 2 summarizes sociodemographic characteristics and the mental health characteristics for all participants overall and stratified by low or high depressive symptoms (PHQ-8 ≥ 10). Mean age was 27.7 (standard deviation (SD) = 5.2), and most participants were female (*n* = 23; 53.5%), non-white (*n* = 26; 60.5%), and single (*n* = 28; 65.1%). The majority of participants had high symptoms of depression (61.9%; PHQ-8 ≥ 10), and a minority had high symptoms of anxiety (37.5%; GAD-7 ≥ 10). A minority were classified as flourishing (16.7%; Flourishing Scale ≥ 48), and fewer than half had high personal recovery (41.9%; Brief INSPIRE-O ≥ 50). Mean scores on both personal recovery and both flourishing measures differed between participants with and without high depressive symptoms. Participants with low depressive symptoms had significantly higher scores on both personal recovery measures and both flourishing measures compared to those with high depressive symptoms (*p* < 0.01).

Table 3 presents the mean and standard deviation of the most important factors in recovery for those with low or high depressive symptoms (PHQ-8 ≥ 10). There were no significant differences between mean importance for each of the factors between those with high and low depressive symptoms. The three items participants ranked as the “most important” factors in their recovery were (1) coping well with stressful events (39.5%), (2) functioning well (34.9%), and (3) having hopes and dreams for the future (30.2%). The lowest rated factors were “feeling happy most of the time” (11.6%) and “absence of symptoms of mental illness,” (18.6%).

**Figure 1 •:**
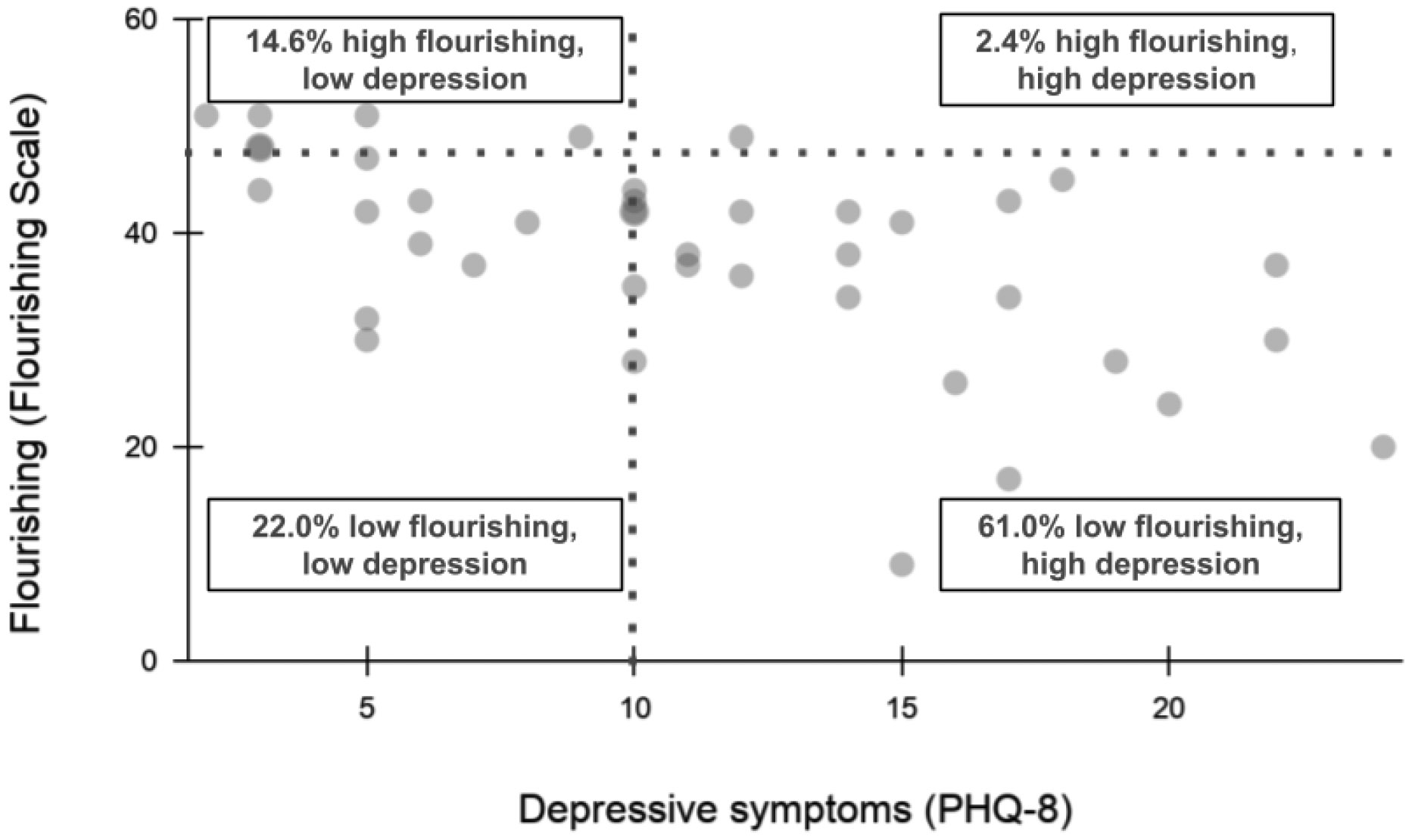
Scatterplot of depressive symptoms and flourishing (*n* = 41). Dotted lines represent standardized cutoffs for Flourishing Scale (≥ 48 represents high flourishing) and Patient Health Questionnaire-8 (PHQ-8; ≥ 10 represents high depression).

**Figure 2 •:**
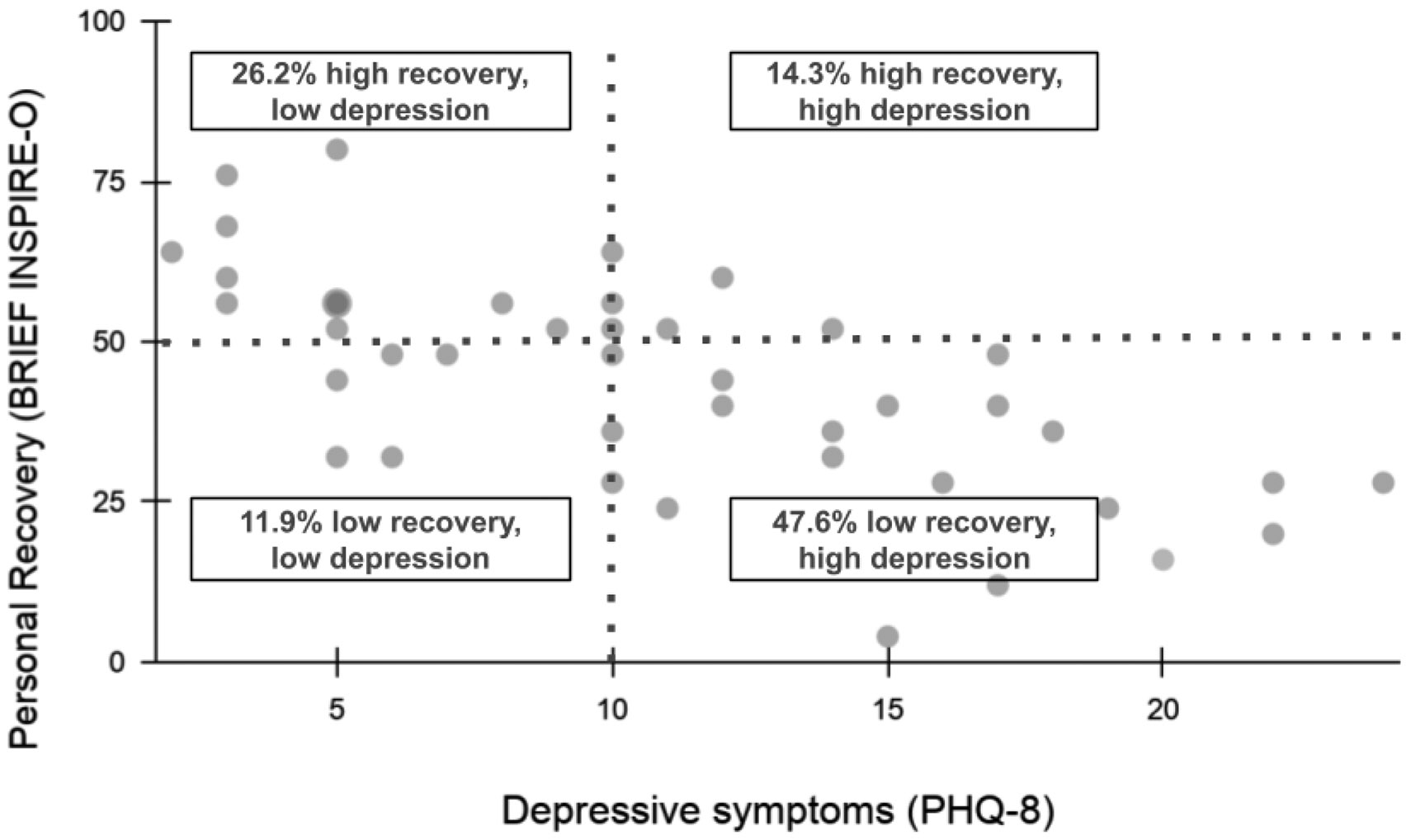
Scatterplot of depressive symptoms and personal recovery (*n* = 42). Dotted lines represent standardized cutoffs for BRIEF INSPIRE-O measure (≥ 50 represents high personal recovery) and Patient Health Questionnaire-8 (PHQ-8; ≥ 10 represents high depression).

Figures 1 and 2 display scatterplots of flourishing (Flourishing Scale ≥ 48 indicating high flourishing) and personal recovery (Brief INSPIRE-O ≥ 50 indicating high personal recovery) in relationship to depressive symptoms (PHQ-8 ≥ 10 indicating high depressive symptoms), divided into the dual-continua model based on cutoff points in the literature. Among individuals with high depressive symptoms (*n* = 26), the vast majority were not flourishing (*n* = 25, 96.1%) and had low levels of recovery (*n* = 20, 76.9%). Among individuals with low depressive symptoms, levels of flourishing and personal recovery were more mixed. Among the individuals with low depressive symptoms who had low levels of “positive” mental health, the majority had low levels of flourishing (*n* = 9/15, 60.0%), and some had low levels of personal recovery (*n* = 5/16, 31.3%). Spearman correlations for the PHQ-8, GAD-7, WSAS, Flourishing Scale, Secure Flourishing Measure, Brief INSPIRE-O, and QPR are displayed in Table S1.

## 4. Discussion

In this diverse sample of young adults with MDD receiving outpatient behavioral health treatment, we found participants valued a broad range of recovery outcomes, with minimal differences between those with high versus low depressive symptoms. Participants were found to exhibit a wide range of depression and “positive” mental health scores, reflecting heterogeneity in participants’ overall mental health. While personal recovery and flourishing were inversely associated with symptoms of depression, a notable proportion of individuals were categorized in the low “positive” mental health/low depressive symptoms quadrant, and the high “positive” mental health/high depressive symptoms quadrant. These findings suggest the importance of assessing “positive” mental health, such as flourishing and personal recovery, alongside symptoms in order to better characterize overall mental health.

This study is among the first to directly elicit from service users with MDD which factors they consider important in recovery. We found that service users with MDD prioritized diverse domains of recovery, including coping well with stress, functioning, and generally feeling good. These results align with a larger shift within psychiatry to increase the focus on assessing functionality, quality of life, and more “positive” outcomes [36]. Despite this shift, current clinical practice and research studies still largely assess symptomatic remission as a primary outcome [18]. Studies demonstrate clinicians often prioritize symptomatic remission over other outcomes [5, 20], which may contribute to misaligned treatment goals. Given treatment alliance is one of the most important predictors of treatment response, aligning goals may improve treatment efficacy [37]. Combining the results of our paper with the previous literature, the heterogeneity of outcomes rated as important suggests the need for tailoring treatment outcomes toward individual patient priorities. Instead of defaulting toward symptomatic remission as the most important goal, clinicians may consider broader conversations about goals of care during the initial assessments.

In this diverse sample treated in an outpatient safety-net behavioral health setting, the mental health of participants spanned a broad range of “positive” mental health and depressive levels. This broad-ranging spectrum of mental health may indicate the need for individualized treatment approaches that extend beyond the current predominant focus on symptomatic reduction. While personal recovery and flourishing were inversely associated with depressive symptoms, the dual-continua model revealed important findings in the inverse quadrant. First, some individuals without significant depressive symptoms nonetheless did not demonstrate high “positive” mental health scores, indicating that low depressive symptoms do not necessarily equate to high characteristics of “positive” mental health. This finding supports the dual-continua model of mental illness whereby a reduction in symptoms and improvement in “positive” mental health can be thought of as two distinct, but related, goals [17]. Second, a subset of participants exhibited flourishing or recovery with significant symptoms of depression. While our pilot study was not designed to test the validity of the “dual-continua model” of mental health, this result is consistent with other larger national survey data in college student samples [38] and demonstrates that even in a sample of individuals with significant depressive symptoms being treated at an outpatient safety-net system, having characteristics of “positive” mental health is achievable. Combining these findings with the findings that individuals care about diverse outcomes, this suggests that behavioral health providers, researchers, and policymakers may be able to both evaluate and intervene on two distinct but related goals: (1) symptomatic reduction and (2) improving “positive” mental health. Previous studies demonstrate characterizing individuals by dual-continua model quadrants may prognosticate future incidence of mental illness better than just assessing symptoms of mental illness [35, 39, 40].

Within the quadrant of individuals without significant depressive symptoms and characteristics of “positive” mental health, a greater proportion of individuals were found to have characteristics of personal recovery compared to characteristics of flourishing. This difference may reflect the origins of the respective measures, with flourishing measures being largely derived from “healthy normal” samples [11], and personal recovery measures being largely derived from individuals with severe mental illness [12], which may have led to differences in standardized cutoff points for the different populations. Personal recovery and flourishing were strongly positively correlated, a finding we could not identify elsewhere in the literature. Despite both of these multidimensional constructs being developed from separate fields, some domains were assessed in both flourishing and personal recovery constructs (e.g., meaning, social relationships, optimism, and feeling generally good) [11, 12]. Future studies will need to assess the psychometric properties, particularly the divergent validity of these constructs.

This study highlights the importance of considering the inclusion of outcome measurement beyond symptomatic remission. Future studies will need to continue to assess how early changes in certain outcome measures (e.g., functionality, symptoms, “positive” mental health, and recovery) may lead to better prognosis for MDD [41].

### 4.1. Limitations

Several limitations are noteworthy. First, this pilot study had a small sample size, limiting the power in our analyses and the ability to compare sub-groups to one another. Secondly, we identified individuals with major depressive disorder by billing codes from electronic health records, which limits the diagnostic certainty of our sample. Additionally, the limited sociodemographic information gathered in our electronic health record screening protocol precluded our ability to systematically assess whether eligible non-participants differed from study participants, potentially introducing selection bias. Moreover, given the delay between electronic health record identification, verbal screening, and survey completion, some of the participants recruited could have taken the survey after they had terminated outpatient mental health treatment. Lastly, although this study was a diverse sample of individuals from a safety-net clinic, the online survey design, which was only provided in English, may have unintentionally excluded participants who were not comfortable completing online surveys or were not fluent in English. The cross-sectional nature of this study limits our ability to understand how personal recovery and flourishing outcomes may change over time. Future longitudinal studies may be able to assess how behavioral health utilization affects “positive” mental health or if “positive” mental health may be protective against future incidence of mental illness. Our study team is also conducting semi-structured qualitative interviews with a subsample of these participants, to better understand these quantitative findings. Researchers and clinicians will need to identify reliable, valid, easy-to-administer, acceptable, transdiagnostic outcome measures in order to better characterize overall mental health.

## 5. Conclusions

Individuals with MDD value a diverse range of outcomes that extend beyond symptomatic remission, and exhibit a large variation in levels of depressive symptoms and characteristics of “positive” mental health. These findings may suggest the need to personalize treatment toward patient-defined goals, which include more “positive” outcomes. Assessing individuals’ symptoms of mental illness alongside “positive” mental health constructs may better characterize overall mental health.

## Supplementary Material

Recruitment flowchart

The supplementary materials are available at https://doi.org/10.20935/MHealthWellB8342.

## Figures and Tables

**Table 1 •:** Measures and descriptions of measures.

	Description	Number of items	Range	Standardized cutoff
“Positive” mental health measures				
Flourishing				
Flourishing Scale [24]	Self-report scale to assess domains of purpose, social relationships, engagement, contribution, competence, self-esteem, optimism, and respect.	8	8–56	≥ 48 indicates flourishing [24]
Secure Flourishing Measure [25]	Self-report measure to assess domains of happiness and life satisfaction, mental and physical health, meaning and purpose, character and virtue, close social relationships, and financial and material stability.	12	0–120	High scores indicate more flourishing; no established cutoff
**Personal recovery**				
Brief INSPIRE-O [26]	Patient-reported outcome measure designed to measure personal recovery domains of connectedness, hope, identity, meaning and empowerment.	5	0–100	≥ 50 indicates “good” personal recovery [27]
Questionnaire about the Process of Recovery [28]	Self-report questionnaire, which measures domains of connectedness, hope, identity, meaning and empowerment.	15	15–75	High scores indicate more personal recovery; no established cutoff
**Symptoms/functioning measures**				
Patient Health Questionnaire-8 [29]	Self-report questionnaire to measure depressive symptoms, including depressed moods or anhedonia based on Diagnostic and Statistical Manual symptom criteria except for suicidal ideation over the last two weeks.	8	0–24	≥ 10 indicates high depressive symptoms [30]
Generalized Anxiety Disorder 7-item scale [31]	Self-report questionnaire to measure anxiety symptoms, including feeling on edge or excessive worrying based on Diagnostic and Statistical Manual symptom criteria over the last two weeks.	7	0–21	≥ 10 indicates high anxiety symptoms [31]
Work and Social Adjustment Scale [32]	Self-report scale assessing impairment across five domains: work, home management, social leisure activities, private leisure activities, and close relationships.	5	0–40	≥ 20 indicates severe functional impairment [32]

**Table 2 •:** Sociodemographic factors and mental health.

	*n* (%) *N* = 43 *	*n* (%) low depressive symptoms *n* = 16	*n* (%) high depressive symptoms *n* = 26	*p* value (low vs. high depressive symptom groups)
**Gender**				
Male	11 (25.6%)	7 (43.8%)	4 (15.4%)	0.047
Female	23 (53.5%)	5 (31.3%)	18 (69.2%)	
Gender expansive	9 (20.9%)	4 (25.0%)	4 (15.4%)	
**Race/ethnicity**				
White	17 (39.5%)	7 (43.8%)	9 (34.6%)	0.47
Black	4 (9.3%)	2 (12.5%)	2 (7.7%)	
Hispanic/Latin(x)	10 (23.3%)	5 (31.3%)	5 (19.2%)	
Asian/Asian American	4 (9.3%)	1 (6.3%)	3 (11.5%)	
Other/multiple	8 (18.6%)	1 (6.3%)	7 (26.9%)	
**Relationship status**				
Single	28 (65.1%)	9 (56.3%)	18 (69.2%)	0.60
Partnered	12 (27.9%)	6 (37.5%)	6 (23.1%)	
Other	3 (7.0%)	1 (6.3%)	2 (7.7%)	
	M (SD)	M (SD)	M (SD)	
**Age**	27.7 (5.2)	29.4 (5.0)	26.7 (5.3)	0.11
**Symptoms/functionality**				
Patient Health Questionnaire-8	11.0 (5.9)	5.0 (1.9)	14.7 (4.2)	<0.01
Generalized Anxiety Disorder-7	8.8 (4.5)	5.9 (2.8)	10.5 (4.5)	<0.01
Work and Social Adjustment Scale	18.5 (10.0)	13.3 (7.6)	21.9 (10.2)	0.01
**Flourishing**				
Flourishing Scale	38.2 (9.6)	43.5 (6.7)	34.8 (9.6)	<0.01
Secure Flourishing Measure	53.1 (19.4)	64.1 (11.7)	46.2 (20.5)	<0.01
**Personal recovery**				
Brief INSPIRE-O	43.9 (16.9)	55.0 (13.3)	36.5 (15.2)	<0.01
Process of Recovery	51.8 (11.3)	60.6 (9.0)	46.6 (9.5)	<0.01

High depressive symptoms defined by PHQ-8 ≥ 10.

* One participant did not have complete PHQ-8 data and was excluded from depression subgroup analyses. *p* value represents chi2 test for categorical data and two-sided t test for continuous data for low vs. high depressive symptom groups. M: mean; SD: standard deviation.

**Table 3 •:** Most important factors in recovery, ranked by highest mean score (*N* = 43).

	Mean (SD)	*n* (%) ‘Most important’
Coping well with stressful events	3.2 (0.8)	17 (39.5)
Functioning well	3.0 (0.9)	15 (34.9)
Having hopes and dreams for the future	3.0 (0.8)	13 (30.2)
Return to usual level of functioning at work, home, school	2.9 (0.9)	15 (34.9)
General sense of well-being	2.9 (0.9)	12 (27.9)
Having positive relationships	2.8 (0.9)	10 (23.3)
Having purpose and meaning	2.8 (0.8)	10 (23.3)
Feeling generally good about yourself	2.7 (1.0)	12 (27.9)
Feeling in control	2.7 (0.9)	9 (20.9)
Participating in and enjoying usual activities *	2.7 (0.8)	8 (19.1)
Feeling satisfied with life	2.6 (1.0)	9 (20.9)
Absence of symptoms of mental illness	2.5 (1.0)	8 (18.6)
Feeling happy most of the time	2.1 (1.0)	5 (11.6)

Importance to personal recovery was rated on a 4-point Likert scale: 1 (“Not very important”)–4 (“Most important”). Mean values reflect average ratings across respondents, with higher scores indicating more perceived importance in recovery.

* One participant did not provide a response for “Participating in and enjoying usual activities.” SD: standard deviation.

## Data Availability

The data supporting the findings of this publication can be made available upon request.
