# Supplementary material for: What outcomes matter in recovery from depression? A pilot study of young adults
Source: Acad Ment Health Well Being. Author manuscript; Available in PMC 2026 Aug 11. (PMC13455757; doi:10.20935/MHealthWellB8342)
Supplement: Supplementary file 1 [file NIHMS2196522-supplement-Recruitment_flowchart.pdf]

Supplementary materials

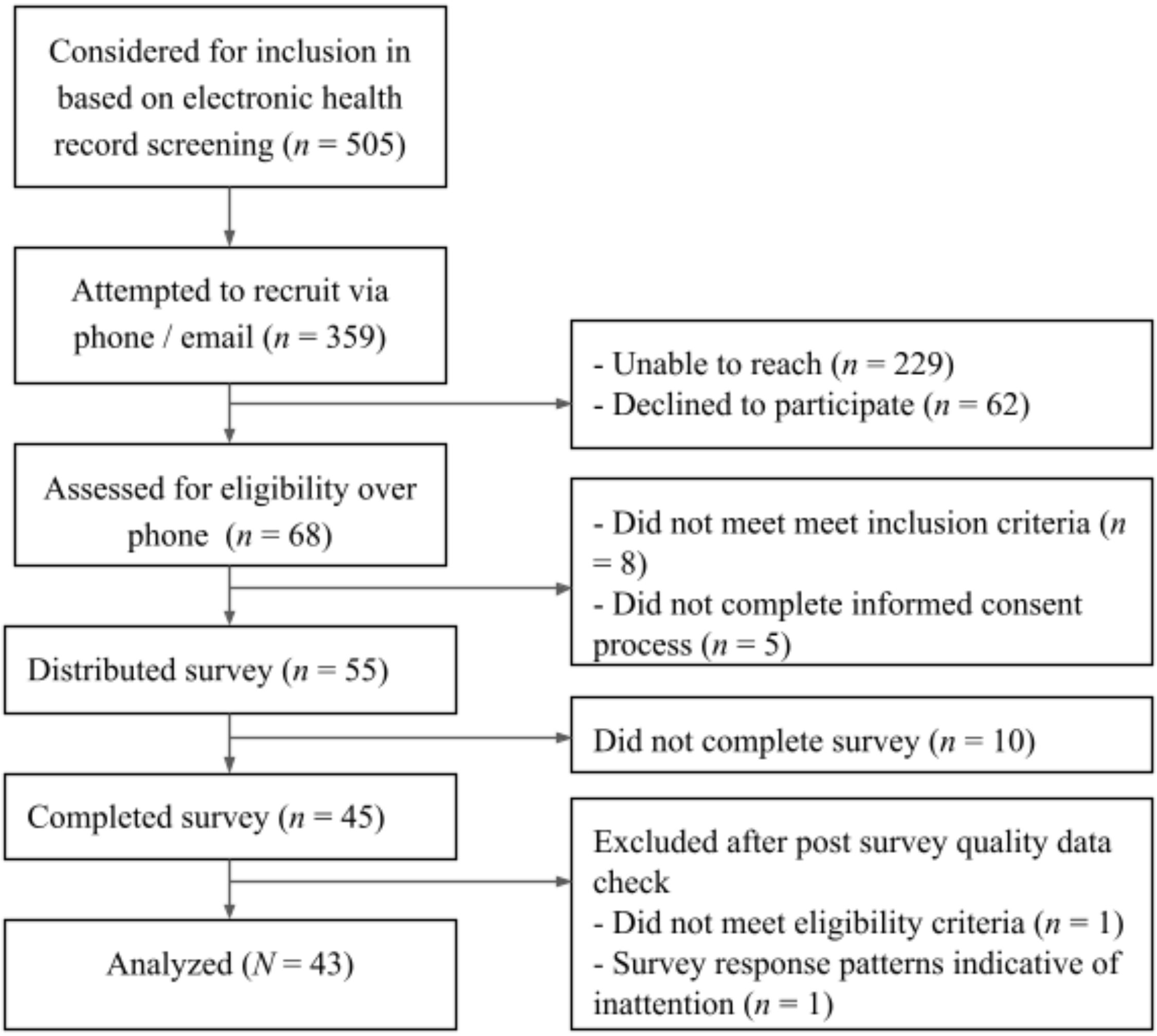

Figure S1. Recruitment flowchart.

Table S1. Descriptive statistics and pairwise correlations.

	n	Cronbach alpha	Patient-Health Questionnaire-8	Generalized Anxiety Disorder-7	Work and Social Adjustment Scale	Flourishing Scale	Secure Flourishing Measure	Brief INSPIRE-O	Process of Recovery
Patient Health Questionnaire-8	42	0.87	1						
Generalized Anxiety Disorder-7	40	0.85	0.71	1					
Work and Social Adjustment Scale	40	0.86	0.62	0.58	1				
Flourishing Scale	42	0.89	−0.59	−0.41	−0.46	1			
Secure Flourishing Measure	42	0.88	−0.61	−0.40	−0.58	0.80	1		
Brief INSPIRE-O	43	0.87	−0.71	−0.50	−0.60	0.81	0.73	1	
Process of Recovery	43	0.92	−0.73	−0.49	−0.55	0.92	0.79	0.79	1

All Spearman correlations were statistically significant at the $p < 0.05$ level.
